# Adaptive divergence generates distinct plastic responses in two closely related *Senecio* species

**DOI:** 10.1111/evo.14478

**Published:** 2022-04-18

**Authors:** Greg M. Walter, James Clark, Antonia Cristaudo, Delia Terranova, Bruno Nevado, Stefania Catara, Momchil Paunov, Violeta Velikova, Dmitry Filatov, Salvatore Cozzolino, Simon J. Hiscock, Jon R. Bridle

**Affiliations:** ^1^ School of Biological Sciences University of Bristol UK; ^2^ School of Biological Sciences Monash University Melbourne Australia; ^3^ Department of Plant Sciences University of Oxford Oxford UK; ^4^ Department of Biological, Geological, and Environmental Sciences University of Catania Catania Italy; ^5^ Center of Ecology, Evolution, and Environmental Changes Universidade de Lisboa Lisboa Portugal; ^6^ Faculty of Biology Sofia University St. Kliment Ohridski Sofia Bulgaria; ^7^ Bulgarian Academy of Sciences, Institute of Plant Physiology and Genetics Sofia Bulgaria; ^8^ Department of Biology University of Naples Federico II Naples Italy; ^9^ Department of Genetics, Evolution, and Environment University College London London UK

**Keywords:** adaptation, adaptive divergence, differential gene expression, environmental sensitivity, evolutionary history, phenotypic plasticity

## Abstract

The evolution of plastic responses to external cues allows species to maintain fitness in response to the environmental variations they regularly experience. However, it remains unclear how plasticity evolves during adaptation. To test whether distinct patterns of plasticity are associated with adaptive divergence, we quantified plasticity for two closely related but ecologically divergent Sicilian daisy species (*Senecio*, Asteraceae). We sampled 40 representative genotypes of each species from their native range on Mt. Etna and then reciprocally transplanted multiple clones of each genotype into four field sites along an elevational gradient that included the native elevational range of each species, and two intermediate elevations. At each elevation, we quantified survival and measured leaf traits that included investment (specific leaf area), morphology, chlorophyll fluorescence, pigment content, and gene expression. Traits and differentially expressed genes that changed with elevation in one species often showed little changes in the other species, or changed in the opposite direction. As evidence of adaptive divergence, both species performed better at their native site and better than the species from the other habitat. Adaptive divergence is, therefore, associated with the evolution of distinct plastic responses to environmental variation, despite these two species sharing a recent common ancestor.

The persistence of species in novel or changing environments often relies on their ability to adjust their phenotype to cope with environmental variation (Chevin et al. 2010). Such phenotypic plasticity generates different phenotypes from the same genotype depending on the environment (Via et al. 1995; Sultan 2000; de Jong 2005). The ability for plasticity to allow species to cope with environmental variation is shaped by selection within environments routinely or historically experienced by populations of that species (Ghalambor et al. 2007). Plasticity is therefore only likely to buffer environmental variation by maintaining fitness when exposed to familiar environmental regimes (Bradshaw 1965; Schlichting 1986; Baythavong and Stanton 2010), which may mean that plastic responses in novel environmental conditions are little help, or are even maladaptive (Chevin and Hoffmann 2017; Hoffmann and Bridle 2021). Understanding how plasticity evolves is therefore important for understanding how species can respond to novel environmental variations (Bradshaw 1965; Baythavong and Stanton 2010).

Plasticity can be thought of as a property of a genotype, individual, and population, as well as at the species level (de Jong 2005; Ghalambor et al. 2007). At the population level, a review of plant reciprocal transplant experiments found that populations often showed no plasticity (i.e., canalised phenotypes were common), and that where plasticity was found, nonadaptive plasticity was more common than adaptive plasticity, suggesting that local adaptation often fails to generate appropriate forms of plasticity (Palacio‐López et al. 2015). It is therefore difficult to understand how or why plasticity evolves during local adaptation. By quantifying how plasticity differs between closely related species that have recently become adapted to contrasting habitats, it is possible to identify how adaptive divergence is associated with differences in plasticity at the species level (Sultan 2001; Murren et al. 2014).

The effect of adaptive divergence on plasticity will depend on how selection interacts with plasticity (de Jong 2005; Radersma et al. 2020). In general, phylogenetic relatedness (Pigliucci et al. 1999; Kellermann et al. 2018), ecology (Kulkarni et al. 2011), and the predictability of the environment (Oostra et al. 2018; Leung et al. 2020) determine the nature and amount of variation in plastic responses, as well as the range of environments within which plastic responses are adaptive (Hoffmann and Bridle 2021). Although contrasting environments are expected to select for differences in plasticity (Schlichting 1986; Donohue et al. 2001; de Jong 2005; Murren et al. 2014), we still have a relatively poor understanding of how plasticity evolves during adaptation. This is because fitness and plasticity are rarely quantified for closely related species from contrasting habitats that are reciprocally transplanted into their native habitats.

At the species level, plasticity evolves when genotypes vary in their sensitivity to environmental variation, and selection favours genotypes with a particular magnitude or direction of plastic changes in phenotype (Via and Lande 1985; Thompson 1991; Oostra et al. 2018). During adaptive divergence, if selection is strong and one environment is invariable or unpredictable, plasticity should be reduced because any variation in phenotype will reduce fitness (species B in blue, **Fig**. **1a**). By contrast, if selection is variable or predictable in space or time, we expect greater plasticity to evolve (species A in orange, **Fig**. **1a**). Given that the plasticity displayed by a given species reflects past selection for particular plastic responses, we could also expect differences in plasticity to evolve between species if they are adapted to contrasting habitats that select for opposite changes in the phenotype (**Fig**. **1b**). Adaptive divergence should, therefore lead, not only to phenotypic divergence between species, but also divergence in plasticity when different environments favour differences in the direction or magnitude of plasticity (de Jong 2005; Ghalambor et al. 2007).

**Figure 1:**
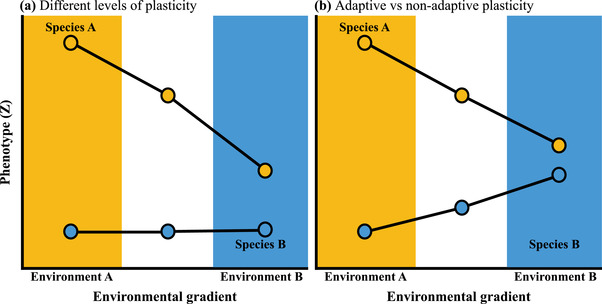
Conceptual diagram depicting the change in a phenotypic trait (Z) across an environmental gradient for two species (A in orange and B in blue) adapted to environments at either end of the gradient. **(a)** Species differ in their magnitude of plasticity: compared to species A, species B shows lower plasticity as little to no change in phenotype across environments, which could be driven by lower variability or predictability in the native environment of species A. (**b)** Species differ in the direction of plasticity and whether plasticity is adaptive: if adapative divergence creates differences in plasticity between two species, then we would expect that the two species will respond differently when exposed to the same environmental variation. In the environment of species B (blue), plasticity moves the phenotype of species A toward the phenotype of species B, suggesting plasticity is somewhat adaptive in that novel environment. By contrast, in the environment of species A (orange), plasticity moves the phenotype of species B away from the native phenotype of species A, suggesting that plasticity in species B is nonadaptive in environment A.

In addition to considering phenotypic traits, estimating variation in gene expression across environments reveals key aspects of the genomic basis of plasticity, and how it differs across ecologically divergent species. Where different allelic (sequence changes in regulatory genes) or epiallelic (e.g., DNA methylation, chromatin remodelling, post‐transcriptional modifications) variants underlying trait plasticity are favoured in different environments, differences in plasticity will arise between species adapting to contrasting environments (Gibson and Wagner 2000; Shaw et al. 2014). If selection varies among environments, divergence in phenotypic plasticity at the gene expression level is expected to occur (Akman et al. 2016).

In this study, we first use a common garden experiment to identify physiological differences between two closely related species of *Senecio* that inhabit contrasting elevations on Mt. Etna, Sicily. We then quantify variation in survival and phenotypic plasticity across an elevation gradient that includes the native habitats of both species. *Senecio chrysanthemifolius* (**Fig**. **2a**) is a short‐lived perennial (individuals generally live for less than 2 years) with highly dissected leaves that occupies disturbed habitats (e.g., vineyards, abandoned land, and roadsides) in the foothills of Mt. Etna about 400–1000 m a.s.l (above sea level) and at similar elevations throughout Sicily. By contrast, *Senecio aethnensis* (**Fig**. **2b**) is a longer‐lived perennial with entire glaucous leaves and is endemic to lava flows about 2000–2600 m a.s.l on Mt. Etna that are covered by snow each winter. Vagrant individuals are often observed at intermediate elevations, suggesting that their distribution can extend to intermediate elevations. These two species are proposed to have diverged recently, possibly about 150,000 years ago, around the same time that the uplift of Mt. Etna created the high elevation environment in which *S. aethnensis* is found (Chapman et al. 2013; Osborne et al. 2013). Their recent shared ancestry is reflected by very low genetic divergence across the genome, despite large differences in habitat, phenotype, and life history (Chapman et al. 2016). In particular, rapid divergence in leaf shape and dissection is a key feature of adaptive divergence between these species (Brennan et al. 2009), as well as in adaptive radiations of other *Senecio* species (Walter et al. 2018; 2020a).

**Figure 2:**
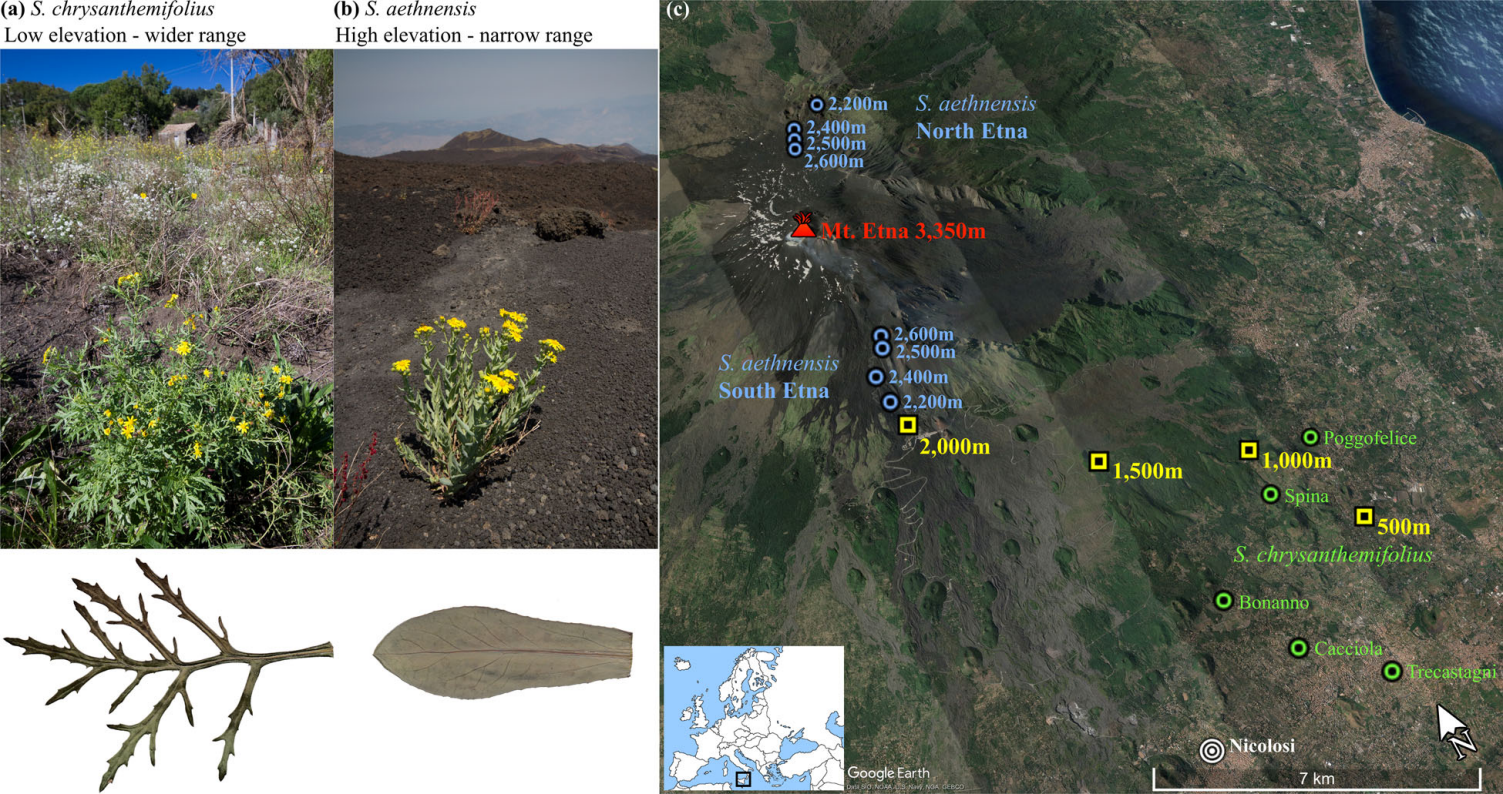
**(a)** *Senecio chrysanthemifolius* occupies disturbed habitats below approximately 1000 m a.s.l, and has thin, dissected leaves. **(a)** *Senecio aethnensis* inhabits lava flows and has thicker, smooth‐margined leaves with a thick waxy cuticle. **(c)** Map of sampling locations (*S. chrysanthemifolius*: green circles; *S. aethnensis*: blue circles) and transplant sites (yellow squares). Sampling locations for *S. aethnensis* and the transplant sites are labelled by their elevation. Inset map shows the location of the study system within Europe.

We predicted that (1) the occupation of contrasting habitats by these species would be reflected by differences in physiology in a common garden. Specifically, we would observe greater water use efficiency and lower leaf pigment content for UV light defence in the low elevation species (*S. chrysanthemifolius*) compared to the high elevation species (*S. aethnensis*). (2) As evidence of adaptive divergence, each species would perform better at their native elevations than beyond their existing range, and better than the other species within their native habitat. (3) Despite their recent common ancestry, as a consequence of adaptive divergence, the two species would show differences in the direction and magnitude of plastic changes in phenotype and gene expression across the elevational gradient. (4) Given that *S. aethnensis* is restricted to high elevations on Mt. Etna while *S. chrysanthemifolius* is distributed throughout lowland Sicily, we expected that *S. chrysanthemifolius* would show greater plasticity across the elevational range than *S. aethnensis* (**Fig**. **1a**); and (5) compared to *S. chrysanthemifolius*, plasticity in *S. aethnensis* would be less adaptive in a novel environment because it would fail to a greater extent to move its phenotype toward the native phenotype when transplanted in the habitat of the other species (**Fig**. **1b**).

To test these hypotheses, we sampled c40 genotypes of *S. chrysanthemifolius* and *S. aethnensis* from natural populations and conducted extensive reciprocal transplant experiments in 2017 and 2019. Propagating genotypes of both species from cuttings allows us to control for genetic variation by assaying the same genotypes at multiple sites using multiple clones per genotype, providing biological replication of phenotypic traits and gene expression profiles. Both experiments involved transplanting cuttings of each genotype to four transplant sites across an elevational range that included the native habitats of both species, and two intermediate elevations where vagrant individuals of both species are often observed. In each transplant experiment, we quantified survival, growth, and leaf traits including leaf shape, gene expression, photosynthetic activity, pigment content, and leaf investment.

## Materials and methods

### SAMPLING NATURAL POPULATIONS

We sampled achenes, referred to hereafter as “seeds,” and took cuttings from naturally growing individuals of both species after plants started flowering. Sampling was conducted in May–June 2017 for *S. chrysanthemifolius* and July 2017 for *S. aethnensis* because *S. aethnensis* grows more slowly and flowers later than *S. chrysanthemifolius*, given its higher elevation habitat. For *S. chrysanthemifolius*, we sampled 37 individuals from five sites between 500 and 800 m a.s.l (**Fig**. **2c**, Supporting information **Table** **S1**). For *S. aethnensis*, we sampled 42 individuals at four different elevations (2600, 2500, 2400, and 2300 m a.s.l) on both the North and South slopes of Mt. Etna (**Fig**. **2c**, Supporting information **Table** **S1**). Although this species occurs at 2000 m, we avoided sampling below 2300 m to avoid the risk of sampling hybrids associated with a stable hybrid zone present at 1500–1700 m (Brennan et al. 2009). To minimise the risk of sampling close relatives, most plants sampled were more than 10 m apart.

We then used the seeds and cuttings sampled from the individuals in the natural populations for three separate experiments. First, to test whether the two species have evolved differences in physiology under common garden conditions, we germinated seeds and grew plants in the laboratory to measure a suite of physiological traits. Second, to test for differences in plasticity, we conducted two field experiments (in 2017 and 2019) that transplanted multiple cuttings of genotypes of both species across an elevational gradient on Mt. Etna.

Genotypes that were propagated for use in the 2017 experiment were sampled directly from natural populations as cuttings, while genotypes propagated for the 2019 experiment were raised in a common garden from seeds collected from natural populations. We used these two different approaches because in 2017, cuttings of each species could only be sampled, propagated, and transplanted at different times due to the delayed growth of *S. aethnensis* at high elevations. This meant that the 2017 experiment did not fully replicate natural growing conditions because we could only transplant both species in the middle of summer, after they finished growing in natural environments. We therefore conducted the 2019 experiment to transplant both species at the same time and during the natural growing period (spring). In 2019, we used plants propagated from seed under common garden conditions to better control for environmental effects.

### PHYSIOLOGICAL DIFFERENCES BETWEEN SPECIES UNDER COMMON GARDEN CONDITIONS

To assess differences in physiology between the species, we grew plants from field‐collected seeds from a representative site of each species (**Fig**. **2c**: Spina for *S. chrysanthemifolius*, 2500 m South Etna for *S. aethnensis*) in a growth cabinet (see Supporting information **Methods** **S1**). From eight maternal families of *S. chrysanthemifolius*, we grew 34 individuals, and from ten maternal families of *S. aethnensis* we grew 41 individuals (about four individuals/family). Seeds were scarified mechanically and placed in petri dishes containing moist filter paper. Seedlings were transplanted into 640 mL pots with standard potting mix. After 2 months of growth, we measured ecophysiology traits known to be associated with elevational gradients (Mierziak et al. 2014; Berardi et al. 2016): leaf pigment content and mechanisms of light defence, which are important as UV becomes stronger at high elevations, and water use efficiency, which is likely to be more important at lower (hot and dry) elevations.

We used a Dualex+ instrument (ForceA, France) to measure the leaf contents: chlorophyll, anthocyanin, and flavonol pigments. Using an LCpro gas analyser (ADC BioScientific, UK), we measured photosynthetic gas exchange. Intrinsic water use efficiency was calculated as the ratio between photosynthesis and stomatal conductance. Chlorophyll fluorescence was measured using an IMAGING‐PAM M‐series chlorophyll fluorometer (Heinz Walz GmbH, Germany). Using output from the fluorometer, we quantified two mechanisms of physiological light defence of leaves (Supporting information **Methods** **S1**): the unregulated [Y(NO)] and regulated [Y(NPQ)] dissipation of heat. To analyse these data, we used a linear model to test for differences in mean between species by calculating a *t*‐statistic and multiplying the *p* value by the number of tests to account for multiple comparisons.

### FIELD TRANSPLANT SITES

Field transplant sites were at four elevations (500, 1000, 1500, and 2000 m a.s.l) along a transect on the south‐eastern side of Mt. Etna (**Fig**. **2c**). The 500 m site was in a garden among fruit trees, the 1000 m site in an abandoned vineyard among *Quercus ilex*, the 1500 m site was located in an apple and pear orchard, and the 2000 m site was on a lava flow from 1983 (with pine trees present). Both sites defined as “native elevations” (500 m for *S. chrysanthemifolius* and 2000 m for *S. aethnensis*) were located less than 1 km from natural populations. Plants of both species were regularly observed at intermediate elevations (above 500 m and below 2000 m), but were never observed within the native range of the other species. There is an elevational transition in soil type from a silty sand at elevations between 500 and 1500 m, to volcanic sand at 2000 m. At each transplant site, four data loggers (Tinytag Plus, Gemini Data Loggers, UK) recorded the temperature hourly. We took three soil samples at each transplant site, which were analysed for 21 variables that included nutrients, salts, and ions (Nucleo Chimico Mediterraneo Laboratories, Catania, Italy). To analyse the soil data, we used Multi‐Dimensional Scaling (MDS) to calculate the scaled distance between the soil samples taken at all transplant sites.

### FIELD TRANSPLANT PROTOCOLS

In the glasshouse (Giarre, Italy), cuttings from all individuals (hereafter, genotypes) were cut into 5 cm stem segments, each possessing two to three leaf nodes. Each smaller cutting was then dipped in rooting plant growth regulator for softwood cuttings (Germon Bew., Der. NAA 0.5%, L. Gobbi, Italy) and placed in a compressed mix of coconut coir and per liter (1:1) in one cell of an 84‐cell tray. All cuttings from each genotype were kept together in one half of a tray and tray positions were randomised regularly. Trays were kept moist and checked regularly for cuttings that successfully produced roots extending out of the bottom of tray. Rooted cuttings of each genotype were randomised into experimental blocks and then transplanted at all four elevations. Cuttings were transplanted into grids of 20 × 7 plants, with 40 cm separating each cutting. The position of cuttings was randomised with respect to genotype (and source population). To prepare the field sites, we cleared the soil of vegetation and debris, and we turned the soil 30 cm deep immediately prior to the transplant.

### 2017 FIELD TRANSPLANT EXPERIMENT

We transplanted 37 *S. chrysanthemifolius* genotypes (*n =* 109 plants/block; *n =* 327 plants/elevation; N = 1308 plants) and 42 *S. aethnensis* genotypes (*n =* 130 plants/block; *n =* 390 plants/elevation; N = 1560 plants) into three experimental blocks at all four elevations. Depending on the number of cuttings that successfully produced roots, we transplanted 6–15 cuttings per genotype at each transplant site (see Supporting information **Table** **S1**). The two species were transplanted at different times (*S. chrysanthemifolius* in June–July 2017; *S. aethnensis* in August 2017) because seasonal constraints meant that sampling from natural populations of *S. aethnensis* was only possible a month after *S. chrysanthemifolius* had been sampled. Following each transplant, cuttings were watered daily for 3 weeks to encourage establishment. We removed 104 cuttings of *S. aethnensis* that died within 3 days due to transplant shock, which meant 2764 plants were included in the experiment (Supporting information **Table** **S2**). To prevent death during high temperatures in July–August (consistently >35°C), we watered cuttings daily during this period, which allowed us to assess the phenotypic responses of genotypes to what were still stressful conditions. We recorded mortality approximately every 2 weeks and measured the phenotypic traits of all plants at a single time point when both species showed substantial post‐transplant growth (November 2017).

### 2019 FIELD TRANSPLANT EXPERIMENT

In 2019, we repeated the 2017 experiment, but transplanted cuttings of genotypes of both species that were grown (from field‐collected seeds) in the glasshouse at the same time, and then transplanted into the same experimental blocks. Using the protocols outlined above, we germinated and grew five seeds collected from multiple genotypes (*S. aethnensis n =* 25; *S. chrysanthemifolius n* = 21) that were sampled from the natural populations in 2017. After 1 week, one seedling from each maternal genotype was chosen at random and placed in 14 cm pots containing standard potting media and left to grow under supplemental lighting (145 μmol m^2^/s 25 W LED tubes; TSA Technology, Italy). When plants reached at about 30 cm high, we removed all branches and propagated cuttings for the transplant using the same protocol as the 2017 experiment. Plants were left to regrow, and we then took a second round of cuttings to increase replication in the transplant experiment. We transplanted the first round of cuttings on 16th April 2019 into three experimental blocks (3 cuttings/genotype/block; *n =* 139 plants/block; N = 1668 plants), and the second round of cuttings on 24th May into one additional experimental block (2 cuttings/genotype/block; *n =* 92 plants/block; N = 368 plants). Overall, we transplanted 2036 plants. Initial watering occurred daily for 3 weeks, after which it was reduced to once per week to maintain a more natural watering regime than the 2017 experiment.

### CHARACTERIZING LEAF MORPHOLOGY AND PIGMENT CONTENT

To characterise leaf morphology, we sampled and pressed young but fully expanded leaves from each cutting once they showed extensive growth (at least 3 months after transplantation). For the 2017 experiment, we sampled three to five leaves per cutting in November, and for the 2019 experiment, we sampled two leaves in September. Leaves were weighed, and then scanned and morphology quantified using the program Lamina (Bylesjo et al. 2008), which generates estimates of leaf area, perimeter, and the number of indentations (leaf serrations and lobes). To estimate the density of indentations along the leaf margin, we standardised the number of indentations by the perimeter. To estimate the leaf complexity, we calculated perimeter^2^ per area, where lower numbers indicate less complex (i.e., more entire) leaves. As a measures of leaf investment, we weighed leaves and calculated Specific Leaf Area $\left(\mathrm{SLA}\;=\frac{leaf\;area}{leaf\;weight}\right)$, where greater values represent larger leaves per unit of dry mass. For the 2019 field experiment, we used a Dualex Scientific + instrument (Force‐A, France) to quantify the concentration of leaf chlorophyll and flavonol pigments. Flavonols are secondary metabolites that help to reduce oxidative stress caused by abiotic (e.g., temperature or light) and biotic (e.g., herbivory) stressors (Mierziak et al. 2014).

### QUANTIFYING CHLOROPHYLL FLUORESCENCE (2017 FIELD EXPERIMENT)

To quantify photosynthetic capacity across elevation for both species, we measured chlorophyll *a* fluorescence, which estimates the efficiency of the photosynthetic response to intense light. We selected five genotypes at random from each species, which were from three sites for *S. chrysanthemifolius* (**Fig**. **2c**: Spina, Trecastagni, and Cacciola) and three elevations for *S. aethnensis* (2400, 2500, and 2600 m). For each genotype, we measured chlorophyll fluorescence on four cuttings at each elevation (*n =* 40 plants/transplant site, total N = 160). We took measurements at two transplant sites each day, completing all four sites within 1 week in October 2017. For each cutting, we measured four leaves, and to temporally replicate measurements, we measured the same cuttings at each site on a second day. To take measurements, we put leaf clips on four leaves of each plant and dark‐adapted the plants for 30 min by covering them with large black plastic containers. We then took fluorescence induction curve measurements for 2 s at 3500 μmol/s/m^2^ photosynthetic photon flux density from each leaf (clip) using a Handy PEA instrument (Hansatech Instruments Ltd., UK). We calculated PI_total_, the total performance of photosystem I and II (Supporting information **Methods** **S1**).

### STATISTICAL ANALYSES OF SURVIVAL AND UNIVARIATE PLASTICITY

We first tested for significant differences in survival across elevation using mixed effects cox proportional hazards models from the *coxme* version 2.2‐14 (Therneau 2012) package in R version 3.6.1 (R Core Team 2019). Survival at the end of the experiment was used as the response variable (censored at the last point of data collection), and we included transplant elevation and species as fixed effects, and genotype and experimental block as random effects (see Eq. [1]).

We then quantified plasticity as the change in leaf morphology, pigment content, and chlorophyll fluorescence across elevation (all leaf traits were first averaged for each clone) using the linear mixed model within the R package *lme4* version 1.1‐23 (Bates et al. 2015)

(1) $$y_{ijklm}=T_{i}\;+S_{j}+T_{i}\times S_{j}+T_{i}\times G_{k\left(j\right)}+B_{l\left(i\right)}+e_{m\left(ijkl\right)}.$$

Changes in the response variable across transplant elevation were modelled by the *j*th species ($S_{j}$) in the *i*th transplant elevation ($T_{i}$) and their interaction ($T_{i}\times S_{j}$), which were all included as fixed effects. Random effects included the interaction between transplant elevation and genotype $T_{i}\times G_{k(j)}$, which accounted for differences among genotypes at each elevation, and experimental block within each environment ($B_{l(i)}$). The residual error variance was captured by $e_{m(ijkl)}$. Separate implementations of Eq. (1) were used for each (normally distributed) univariate response variable of interest ($y_{ijklm}$). For each implementation of Eq. (1), we tested the significance of the interaction between transplant site and species using likelihood ratio tests. To correct for multiple comparisons, we adjusted the *p* value by multiplying by the number of tests and keeping α = 0.05. To test whether differences in morphology between transplant sites were significant for each species, we used *emmeans* version 1.4 (Lenth 2019) to conduct pairwise *t*‐tests adjusted for multiple comparisons.

### ESTIMATING MULTIVARIATE PLASTICITY

To quantify multivariate plasticity across elevation, we mean standardised each morphological trait (divided by its mean). We then used a multivariate analysis of variance (MANOVA) to test for significant differences in multivariate mean phenotype across elevation and between species. We analysed each experiment separately by including the morphological traits as a multivariate response variable. We included species, transplant elevation, and their interaction as main effects. We included the interaction between transplant site and genotype as the error term, which is the appropriate denominator to calculate *F*‐ratios for the main effects because it tests whether differences among species and transplant elevations are significantly greater than differences among genotypes. To visualise multivariate differences, we used the output of the MANOVA to calculate the D‐matrix (Supporting information **Methods** **S1**), which represents differences in mean multivariate phenotype across species and elevation. We also quantified how the two species differed in their direction of plasticity in response to elevation by calculating the vector of plasticity ($\Delta \bar{x}$) for each species using

(2) $$\Delta {\bar{x}}_{plasticity}={\bar{x}}_{native}-{\bar{x}}_{novel},$$

where $\Delta \bar{x}$ is the vector (standardized to unit length) that represents the change in multivariate phenotype from the native site (${\bar{x}}_{native}$) to the novel elevation (${\bar{x}}_{novel}$), for each species separately (Noble et al. 2019; Radersma et al. 2020). Calculating the angle that separates $\Delta {\bar{x}}_{plasticity}$ for each species quantifies the difference in the direction of plasticity between species. To test whether each species showed adaptive plasticity, we used Eq. (2) to calculate the differences in phenotype between the two species in their native habitats $\left(\Delta {\bar{x}}_{divergence}={\bar{x}}_{S.\;chrysanthemifolius\;\mathrm{at}\;500m}-{\bar{x}}_{S.\;aethnensis\;\mathrm{at}\;2,000m}\right)$. We predicted that if plasticity of a given species was adaptive, then plasticity would move the phenotype in the direction of the native phenotype, which would mean that the vector of plasticity would align with the vector representing phenotypic differences between the species (Radersma et al. 2020).

### SAMPLING OF PLANT TISSUE AND RNA EXTRACTION

To quantify gene expression variation across elevation and between species, we sampled young leaves from 12 genotypes of each species. For each genotype, we collected three to four newly emerged leaves (15–20 mm in length) from three cuttings at each transplant site (2 species × 12 genotypes × 3 clones × 4 elevations; total N = 288) in summer (22–26 July 2019), 86–90 days after the initial transplant. Young leaves were selected to standardise developmental stage across samples, and because high‐quality RNA is difficult to extract from older tissue. All leaves for a cutting were placed in an Eppendorf tube and stored in RNAlater at −20°C. We extracted RNA from each sample using QIAgen RNeasy kits. Library preparation and 3’ QuantSeq RNA sequencing was performed at the Oxford Genomics Centre on an Illumina NextSeq platform, producing 75 bp single‐end reads.

### QUANTIFYING DIFFERENTIAL EXPRESSION ACROSS TRANSPLANT SITES, GENOTYPES, AND SPECIES

A reference transcriptome was assembled for each species (Supporting information **Methods** **S1**). Trimmed reads were mapped to each species’ reference transcriptome using *Salmon* version 0.13.1 (Patro et al. 2017). Quantified read counts were imported using *txImport* (Soneson et al. 2015). Transcripts were initially filtered such that those with <5 mapped reads in 50% of samples were discarded. This, however, excludes transcripts that may have high expression in one species but very low in the other. Instead, if the low mapping rates were predominantly found within a single species (>75%), they were retained. All estimates were repeated using *DESeq2* (Love et al. 2014) and *limma/voom* (Law et al. 2014) according to

(3) $$\begin{array}{ccc} Counts\; & ∼ & \;Species\;+\;Transplant\;Site\;+\;Species\;\\ & & \times \;Transplant\;Site\;+\;Genotype. \end{array}$$

In *limma/voom*, the genotype was modelled as a random effect. For comparisons within species, each treatment was compared with the home transplant site of each species, with differentially expressed genes determined based on an adjusted *p* value <0.01 (Benjamini and Hochberg 1995).

Reference transcriptomes were annotated (Supporting information **Methods** **S1**), and to identify functional categories of differentially expressed genes, gene ontology (GO) enrichment analyses were performed using *topGO* version 2.3.6 (Alexa and Rahnenführer (2019). Enrichment was determined using Fisher's exact test using genes that were significantly differentially expressed (adjusted *p* values < 0.01) between the native elevation and the furthest transplant site.

### WEIGHTED NETWORK CONSTRUCTION OF DIFFERENTIALLY EXPRESSED GENES

Weighted Gene Coexpression Network Analysis identifies correlations of expression among all genes and then forms modules of coexpressed genes within that network. Consensus modules were constructed for each species and visualised using Cytoscape (Shannon et al. 2003), with the expression state of the genes included as metadata (Supporting information **Methods** S1). Each module was then summarized using its first principal component as a module eigengene, which represents the expression profile of the module. We tested for a correlation between each module eigengene in each species and transplant elevation. Each module was tested for GO enrichment in *topGO*, using Fisher's exact test.

## Results

### PHYSIOLOGICAL DIFFERENCES BETWEEN SPECIES UNDER COMMON GARDEN CONDITIONS

**Figure 3:**
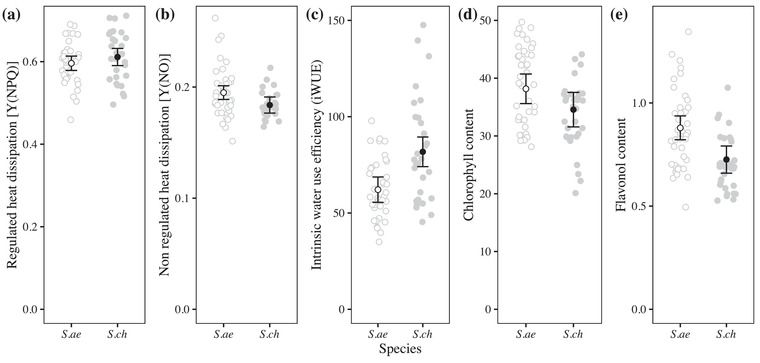
Physiological differences between species grown from seeds under common garden conditions in the laboratory. Filled circles represent *S. chrysanthemifolius* (*S.ch*), while unfilled circles represent *S. aethnensis* (*S. ae*). Grey circles represent individual plants measured and credible intervals represent the 95% confidence intervals of the mean. **(a)** Both species showed similar values for regulated heat dissipation, Y(NPQ). **(b)** *Senecio aethnensis* showed greater values for the unregulated dissipation of heat, Y(NO). **(c)** *Senecio chrysanthemifolius* showed higher intrinsic water use efficiency, while *S. aethnensis* showed higher leaf chlorophyll content **(d)** and a higher flavonol content **(e)**.

Under common garden conditions, both species show a similar ability to regulate heat dissipation from their leaves [Y(NPQ)] (**Fig**. **3a**; t(65) = 0.0116, adj. *p* = 1.000), while *S. aethnensis* showed a greater (but nonsignificant ability for nonregulated heat dissipation [Y(NO)] (**Fig**. **3b**; t(65) = 2.351, adj. *p* = 0.1085). *Senecio chrysanthemifolius* showed evidence of higher intrinsic water use efficiency than *S. aethnensis* (**Fig**. **3b**; t(69) = 3.875, adj. *p* = 0.0010). *Senecio aethnensis* showed slightly greater (but nonsignificant) leaf concentrations of chlorophyll (**Fig**. **3c**; t(143.8) = 2.085, adj. *p* = 0.1940), and greater concentrations of flavonols (**Fig**. **3d**; t(200.9) = 4.399, adj. *p* < 0.0001) than *S. chrysanthemifolius*.

### CHARACTERIZING ENVIRONMENTAL DIFFERENCES ACROSS ELEVATION

**Figure 4:**
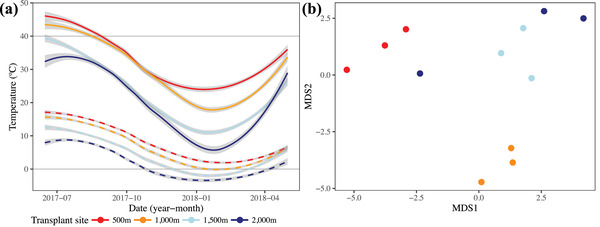
Differences in environment for the four transplant sites at four elevations. **(a)** Average daily maximum (solid lines) and minimum (dashed lines) temperature for three data loggers at each site, for the duration of the transplant. Grey shading represents one standard error in estimating the curves. Higher elevations remained below 40°C in the summer and dropped well below zero in the winter. **(b)** Differences in soil composition for 21 soil variables captured by a multidimensional scaling analysis (Supporting information **Table** **S3** presents the soil data).

The transplant sites experience contrasting climatic conditions associated with elevation, with extreme heat (regularly exceeding 40°C) at 500 and 1000 m during summer, and extreme cold (regularly below 0°C) at 1500 and 2000 m during winter (**Fig**. **4a**). Soil profiles roughly separated the four transplant sites in a linear fashion along the first axis (MDS1), which represented a transition in soil type as a reduction in nutrients (amount of organic material, total nitrogen, cation exchange capacity, and exchangeable ions) at higher elevations (**Fig**. **4b**; Supporting information **Table** **S3**). The second axis (MDS2) characterised differences between the 1000 m site and the other sites, associated with greater concentrations of various salts at 1000 m (soluble nitrates, calcium, and magnesium; Supporting information **Table** **S3**).

### SURVIVAL OF TRANSPLANTED CUTTINGS REFLECTS ADAPTIVE DIVERGENCE BETWEEN SPECIES

**Figure 5:**
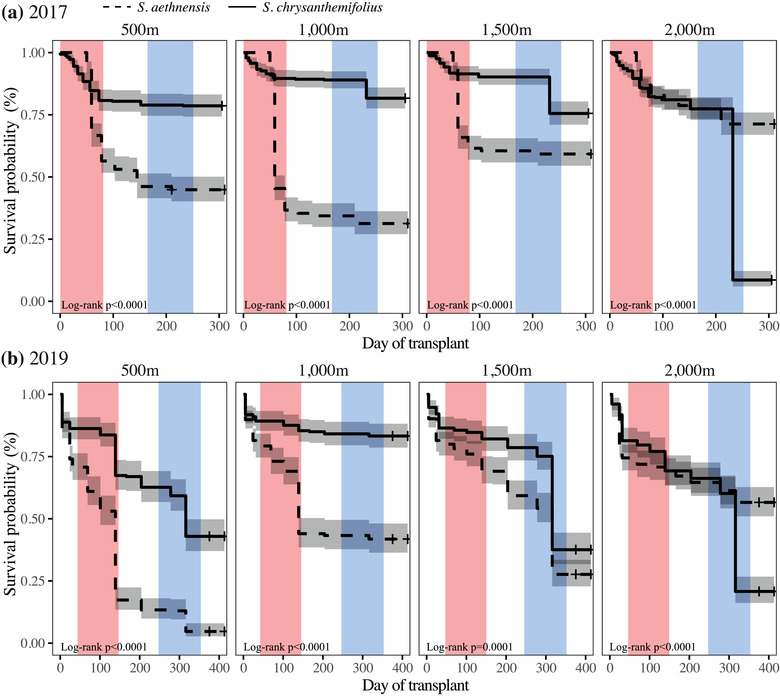
Differences in survival between species across all transplant elevations, for **(a)** 2017 and **(b)** 2019. Solid lines represent *S. chrysanthemifolius* and broken lines represents *S. aethnensis*. Grey shading represents the 95% confidence interval around the mean survival of each species. Shading represents the timing of summer (red) and winter (blue). *Senecio chrysanthemifolius* showed the lowest survival at the highest elevation in both experiments, while *S. aethnensis* showed lower survival at all three lower elevations.

Both experiments showed strong evidence for adaptive divergence between species, in which each species survived consistently better at their home (“native”) site than the species from the other elevation extreme (**Fig**. **5a,b**). Evidence of adaptive divergence was supported by a significant species × elevation interaction for both experiments (2017: *χ^2^* (3) = 27.58, *p* < 0.00001, and 2019: *χ^2^* (3) = 237.55, *p* < 0.00001). *Senecio chrysanthemifolius* showed significantly greater mortality at higher elevations away from its native 500 m site (**Fig**. **5a,b**; Supporting information **Table** **S4**). By contrast, *S. aethnensis* showed significantly greater mortality at lower elevations away from its native 2000 m site (**Fig**. **5a,b**; Supporting information **Table** **S4**). Furthermore, *S. aethnensis* showed greater mortality in summer immediately following the initial transplant, whereas *S. chrysanthemifolius* only showed greater mortality at the highest elevation and only toward the end of the experiment after the winter snow. One exception to this is that in 2019, *S. chrysanthemifolius* suffered about 35% greater mortality at 500 m (its native site) than at 1000 m (which is within the native range). This is likely to be due to newly planted cuttings suffering under intense heat without well‐developed root systems, especially given they received less supplemental water compared to 2017. However, mortality in 2019 was still about 20% greater at the novel elevation (2000 m) than at 500 m, indicating better performance at the native site.

### SPECIES DIFFERENCES IN PLASTICITY: UNIVARIATE TRAIT CHANGES ACROSS ELEVATION

**Figure 6:**
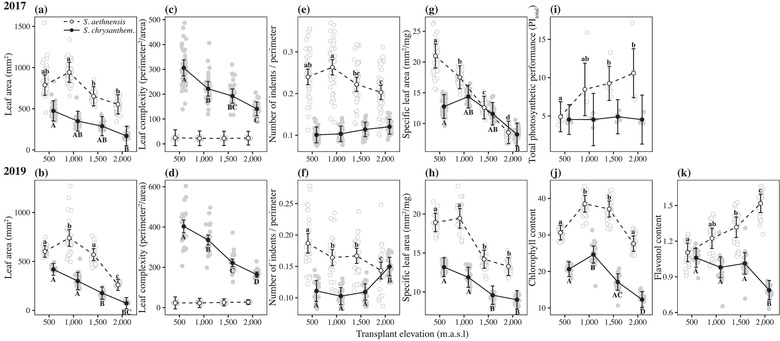
Changes in univariate leaf traits across elevation for both species measured in 2017 (top row) and 2019 (bottom row). Filled circles, solid lines, and upper‐case letters represent *S. chrysanthemifolius*, while unfilled circles, dashed lines, and lowercase letters represent *S. aethnensis*. Credible intervals represent 95% confidence intervals for the estimate of the mean of each species at each elevation. Letters denote significant differences between transplant sites calculated using pairwise tests conducted within each species and adjusted for multiple comparisons (full statistical summaries are given in Supporting information **Table S4**). Grey points represent the mean of all cuttings for each genotype. Traits include: **(a‐b)** leaf area, **(c‐d)** leaf complexity, **(e‐f)** number of indents, **(g‐h)** specific leaf area, **(i)** total photosynthetic performance, **(j)** chlorophyll content, and **(k)** flavonol content. Results are consistent across the 2017 and 2019 experiments, and show that the two species display distinct plastic responses across elevation for leaf complexity, number of indents, specific leaf area, photosynthetic performance, and flavonol content.

As evidence of phenotypic divergence, the two species showed differences in mean phenotype for most traits, regardless of elevation (**Fig**. **6**). Both species also showed phenotypic plasticity as a change in trait mean with elevation for most leaf morphology traits, with patterns of plasticity that were consistent across the 2017 and 2019 experiments (**Fig**. **6**). A significant species × elevation interaction provides strong evidence that the two species change their mean phenotype differently across elevation, and therefore, that they show differences in plasticity. The two species differed in plasticity across elevation, but the extent of differences in plasticity depended on the trait. Both species showed a similar response in leaf area, whereby leaves were larger at lower elevations (**Fig**. **6a,b**; species × elevation in 2017: *χ^2^* (3) = 8.94, adj. *p* = 0.1202, and 2019: *χ^2^* (3) = 42.08, adj. *p* < 0.0001). *Senecio chrysanthemifolius* showed a reduction in leaf complexity at higher elevations, contrasting with no change across elevation in *S. aethnensis* (**Fig**. **6c,d**; species × elevation in 2017: *χ^2^* (3) = 29.09, adj. *p*< 0.0001, and 2019: *χ^2^* (3) = 83.87, adj. *p* < 0.0001). By contrast, *S. aethnensis* showed a reduction in leaf indentation at higher elevations, while *S. chrysanthemifolius* showed no significant change in leaf indentation across elevation in 2017, but a significant increase in leaf indentation at 2000 m in 2019 (**Fig**. **6e,f**; species × elevation in 2017: *χ^2^* (3) = 29.07, adj. *p* < 0.0001, and 2019: *χ^2^* (3) = 66.68, adj. *p *< 0.0001). Both species also showed a similar increase in SLA at lower elevations, but *S. aethnensis* showed a much greater increase at 500 and 1000 m (**Fig**. **6g,h**; species × elevation in 2017: *χ^2^* (3) = 21.95, adj. *p* = 0.00027, and 2019: *χ^2^* (3) = 37.71, adj. *p* < 0.0001), suggesting that *S. aethnensis* produced larger leaves per unit mass than *S. chrysanthemifolius* at lower elevations (and at all elevations in 2019).

In 2017, we measured chlorophyll fluorescence to calculate the total performance index (PI_total_), which estimates the capacity of the photosynthetic machinery. Although *S. chrysanthemifolius* showed no change in PI_total_ across elevation, *S. aethnensis* showed a steady decline at lower elevations and about 50% reduced photosynthetic activity at the lowest elevation (**Fig**. **6i**; species × elevation *χ^2^* (3) = 24.59, *p* < 0.0001). In 2019, we measured the concentration of chlorophyll and flavonols in leaves. Compared to *S. chrysanthemifolius, S. aethnensis* showed higher concentrations of both pigments. Both species showed the highest concentration of chlorophyll at 1000 m, which decreased at higher elevations (and at 500 m for *S. aethnensis*) (**Fig**. **6j**; species × elevation: *χ^2^* (3) = 64.23, adj. *p* < 0.0001). However, the two species show contrasting patterns of plasticity in flavonol content across elevation (**Fig**. **6k**; species × elevation: *χ^2^* (3) = 98.57, adj. *p* < 0.0001): although both species show similar values at 500 m, flavonol content in *S. chrysanthemifolius* decreases at higher elevations, while in *S. aethnensis* it increases at higher elevations.

### SPECIES DIFFERENCES IN PLASTICITY: CHANGES IN MULTIVARIATE PHENOTYPE ACROSS ELEVATION

**Figure 7:**
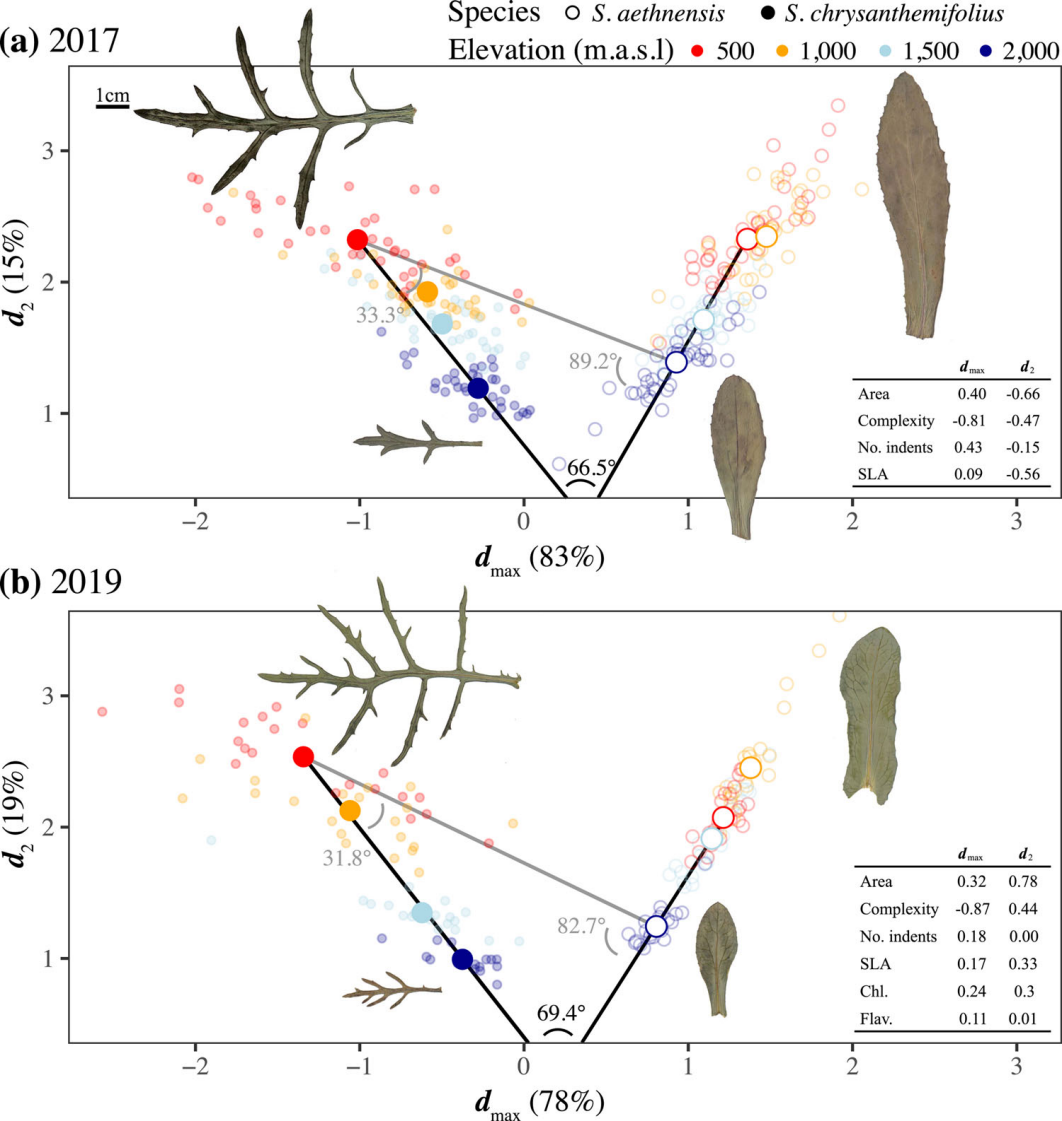
Multivariate analysis (MANOVA used to calculate the D‐matrix) of leaf morphology across elevation for both species measured in **(a)** 2017 and **(b)** 2019. For both experiments, ***d***_max_ represents a trade‐off between leaf size and complexity, and separates both species. By contrast, ***d***_2_ represents similar elevational changes in most traits. Small, filled circles represent genotypes of *S. chrysanthemifolius*, and unfilled circles represent *S. aethnensis*. Large circles represent the multivariate mean at each elevation. Table inset shows the trait loadings for both multivariate axes. Inset leaf images represent a genotype of each species with a value close to the multivariate mean at the elevational extremes (500 and 2000 m). Black lines represent the vector of plastic responses ($\Delta \bar{x}$) across elevation for each species. The angle between plasticity vectors show that the two species differ in the direction of plasticity by 66–69**°**. If plasticity in novel environments was adaptive, we would expect plasticity to occur in the direction of the phenotype of the native species, represented by the vector of phenotypic divergence between the species (grey lines). Angles in grey show that plasticity in *S. chrysanthemifolius* was in a similar direction to phenotypic divergence (31–33**°**), whereas plasticity in *S. aethnensis* was in a different direction (82–89**°**).

To estimate multivariate plasticity, we quantified variation in leaf morphology between species and across elevation by analysing all leaf traits using a MANOVA. For both experiments, we found significant species × elevation interactions (**Fig**. **7**; 2017: Wilks’ λ= 0.375, *F*_3,298_ = 29.19, *p* < 0.0001; 2019: Wilks’ λ= 1.330, *F*_3,176_ = 22.97, *p* < 0.0001). In both experiments, the first axis of **D** (***d***_max_) described >75% of the difference in multivariate phenotype, which primarily separated the two species, generated mainly by a negative correlation between leaf area and complexity (larger, simple leaves versus smaller, more complex leaves). The second axis (***d***_2_) described 15–19% of the difference in multivariate phenotype, which captured differences across elevation as reductions in most traits at higher elevations. The vectors representing elevational plasticity ($\Delta {\bar{x}}_{plasticity}$) differed between species by 66.5**°** and 69.4**°,** respectively for the 2017 and 2019 experiments (black lines in **Fig**. **7**), which suggests the two species showed plastic responses to elevation that differ consistently in direction across both years. For *S. aethnensis* outside its native range (i.e. at 500 m), the vector representing plasticity was in a different direction to that of the native phenotype of *S. chrysanthemifolius* (2017 = 89.2**°**; 2019 = 82.7**°**; **Fig**. **7**). By contrast, for *S. chrysanthemifolius* at the novel 2000 m elevation, plasticity aligned quite closely with the native phenotype of *S. aethnensis* in both experiments (2017 = 33.3**°**; 2019 = 31.8°; **Fig**. **7**). This suggests that *S. chrysanthemifolius* showed strong evidence of adaptive plasticity, while *S. aethenensis* showed more evidence of nonadaptive plasticity.

### DIFFERENTIAL GENE EXPRESSION BETWEEN SPECIES

**Figure 8:**
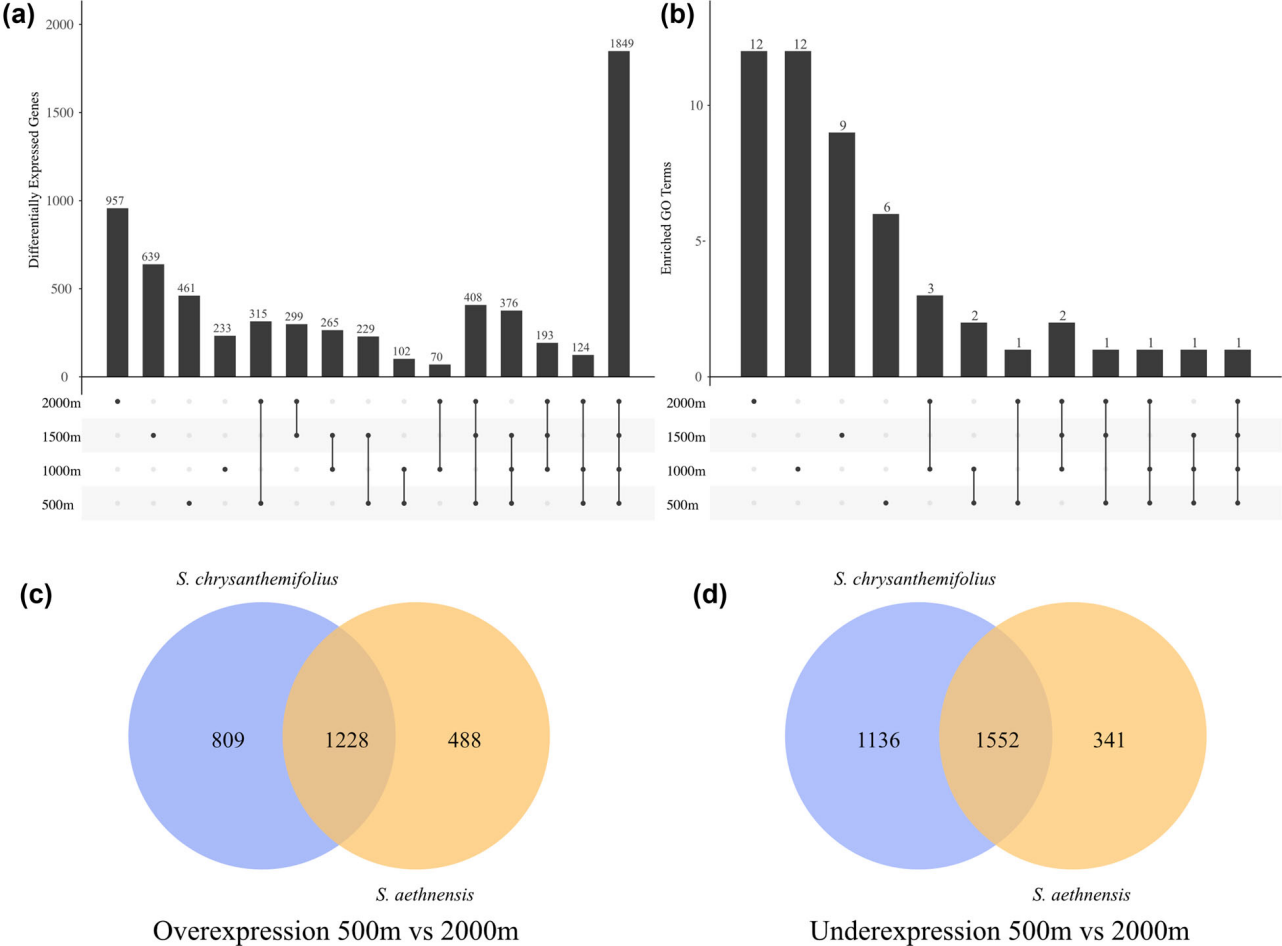
Contrasting patterns of gene expression plasticity between species. (**a**) Total numbers of differentially expressed genes between species at each transplant site. Bars represent the total number of genes in each elevation or set of elevations, which are denoted by the dots below. Many genes that were differentially expressed between the species were unique to each elevation. **(b)** Total numbers of significantly enriched (Fisher's exact test, *p* < 0.05) GO terms between species at each transplant site. Bars represent the total number of GO terms in each elevation or set of elevations, which are denoted by the dots below. Most significant functional categories were specific to each elevation, which were rarely shared across elevations. (**c**) Overlapping overexpressed and underexpressed genes between the home and furthest transplant site in each species. Across elevation, *S. chrysanthemifolius* underexpressed and overexpressed more genes than *S. aethnensis*.

Gene expression patterns for the 2019 transplant revealed strong differences in gene expression between species as well as across elevation (Supporting information **Figs**. **S1**‐**2**). At any given transplant site, more than 200 genes were differentially expressed between the species (**Fig**. **8a**). A total of 1849 genes were differentially expressed between species at all transplant sites, which represents 43–58% of the total number of differentially expressed genes at each site. Therefore, almost half the genes that were differentially expressed between species were consistent across elevation. More genes were differentially expressed between the species at higher elevations (1500 m = 639 genes; 2000 m = 957 genes) compared to lower elevations (500 m = 461 genes; 1000 m = 233 genes) (**Fig**. **8a**). Similarly, functional enrichment of differentially expressed genes between the species shows that more significantly enriched GO terms were unique to 2000 m (12 terms), compared to 500 m (6 terms) (**Fig**. **8b**). Only one GO term was significantly enriched between species at all transplant sites (**Fig**. **8b**), which suggests that although many genes are consistently differentially expressed between the two species, they are not enriched for specific functions.

### DIFFERENTIAL GENE EXPRESSION ACROSS ELEVATION WITHIN SPECIES

Both methods of estimating differential expression (*DESeq2* vs. *limma/voom*) produced similar patterns, which showed more genes were differentially expressed as genotypes of both species were moved further from their native elevations (Supporting information **Fig**. **S2**). *Senecio chrysanthemifolius* differentially expressed more loci between the extreme elevations (4725 out of c.19,000 loci) compared to *S. aethnensis* (3609 loci). In addition, roughly half of the genes that were differentially expressed between the native site and the most novel environment (i.e., the elevational extremes) were unique to each species (**Fig**. **8c,d**), providing evidence that the two species show distinct patterns of plasticity in gene expression. Compared to *S. aethensis, S. chrysanthemifolius* both overexpressed (**Fig**. **8c**) and underexpressed (**Fig**. **8d**) more genes.

**Figure 9:**
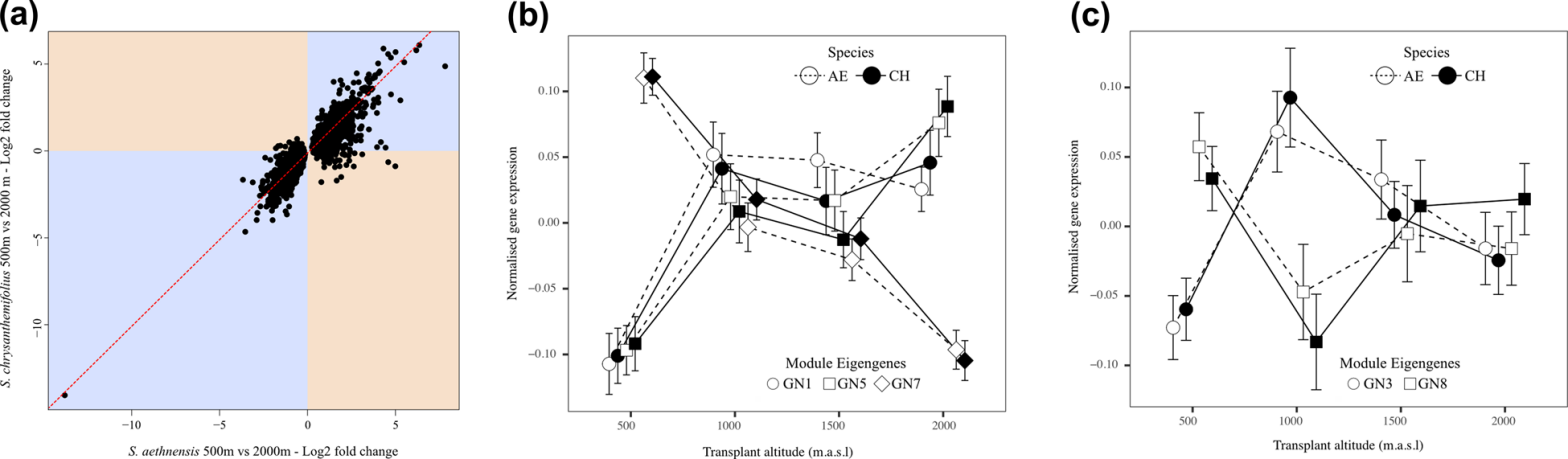
Species differed in their gene expression responses to elevation. **(a)** For most genes, strong over‐ or underexpression across elevational extremes typically showed the same response in both species (blue quadrants). However, for about 50% of differentially expressed genes, the two species differed in the magnitude of gene expression change (blue quadrants: genes that deviate from the red line), and for 121 genes, they showed opposite responses in expression (orange quadrants). **(b‐c)** Normalized expression profiles of module eigengenes across all elevations for *S. chrysanthemifolius* (solid lines and circles) and *S. aethnensis* (dashed lines and unfilled circles). This includes the eigengenes (represented by different shapes) that were: **(b)** strongly correlated with elevation in each species; and **(c)** were significantly correlated with elevation in *S. aethnensis*, but not in *S. chrysanthemifolius*.

Although most of the differentially expressed genes showed responses to elevation that were in a similar direction for both species, 121 (of c.4000) genes showed responses that differed in direction between the species: strong overexpression or underexpression in one species contrasted with relatively unchanged expression across elevation in the other species (**Fig**. **9a**, genes in orange quadrants). Furthermore, about 50% of all differentially expressed genes showed differences between the species in the magnitude of gene expression response to elevation (**Fig**. **9a**, genes away from red line in blue quadrant). Functional enrichment analyses of differentially expressed genes between the elevational extremes revealed 11 significant GO terms in both species. Only two functional categories of these genes were shared between species, again suggesting species‐specific responses on a functional level (Supporting information **Tables** **S5** and **S6**).

Network reconstruction generated 13 network modules ranging in size from 185 to 3345 genes (Supporting information **Table** **S7**, **Fig**. **S3**). In six modules, the module eigengene correlated with elevation in both species (**Fig**. **9b**), while two modules (Supporting information **Table** **S7** and **Fig** **S3**: GN3 and GN8) showed a correlation with elevation unique to *S. aethnensis* (**Fig**. **9c**). No gene modules that correlated with elevation were specific to *S. chrysanthemifolius*. The two modules that changed in *S. aethnensis* but not in *S. chrysanthemifolius* were associated with functions related to pathogen detection and responses to light intensity and UV.

## Discussion

Given that the two *Senecio* species on Mt. Etna are the result of recent ecological divergence (Chapman et al. 2013), we predicted that (1) the two species would show differences in physiology when grown in common garden conditions. (2) They would perform better at their native elevations than beyond their existing range, and better than the other species within their native habitat. (3) They would display differences in plasticity at the phenotypic and gene expression levels. (4) As a more widespread species, *S. chrysanthemifolius* would show plasticity of a greater magnitude compared to *S. aethnensis* (**Fig**. **1a**); and (5) compared to *S. chrysanthemifolius*, plasticity in *S. aethnensis* would be less adaptive because it would struggle to a greater extent to approach the phenotype of the native species when transplanted at the novel elevation (**Fig**. **1b**).

Consistent with our first prediction, the two species showed distinct behaviour in the common garden experiment (**Fig**. **3**), indicating that adaptation to contrasting habitats has generated substantial differences in physiology. Supporting our second prediction, both species performed better at their native site than the other species. Each species also tended to perform best at their native site compared to other elevations, with substantial reductions in survival observed at the elevation furthest from their native site (i.e., at the native site of the other species; **Fig**. **5**). This provides strong evidence that these two species are each adapted to their different habitats, which is consistent with another field experiment that demonstrated adaptive divergence between these two species when transplanted as seeds (Walter et al. 2021). Consistent with our third prediction, we found different patterns of plasticity in phenotype (**Figs**. **6**, **7**) and gene expression profiles (**Figs**. **8**, **9**) between the two species. In support of our fourth prediction, *S. chrysanthemifolius* over‐ and underexpressed more genes across elevation than *S. aethnensis* (**Fig**. **8c,d**), suggesting a greater magnitude of plasticity for *S. chrysanthemifolius*. For the leaf traits, however, we found that the extent of differences in plasticity between the two species depended on the trait (**Fig**. **6**). Consistent with our fifth prediction, plasticity was more adaptive in *S. chrysanthemifolius* than *S. aethnensis*: Plasticity moved the phenotype of *S. aethnensis* away from the native phenotype of *S. chrysanthemifolius* at 500 m, whereas plasticity moved the phenotype of *S. chrysanthemifolius* closer to the native phenotype of *S. aethnensis* at 2000 m (**Fig**. **7**).

### ADAPTIVE DIVERGENCE TO ELEVATIONAL EXTREMES

In the common garden and field experiments, the two species show large differences in mean phenotype, which is likely associated with adaptation to their contrasting environments. In the common garden experiment, greater flavonol content (also observed in the field experiment) and unregulated light defence in *S. aethnensis* are likely to reflect a more constant need for light defence in the high elevation habitat where UV levels are greater (Mierziak et al. 2014; Berardi et al. 2016). Glaucous leaves with large smooth margins is also likely to be an adaptation that helps *S. aethnensis* to cope with the harsh high‐elevation habitat, whereas more complex leaves, such as those of *S. chrysanthemifolius*, are associated with greater heat tolerance that will likely be required at low elevations (Royer et al. 2009).

### ADAPTIVE DIVERGENCE CREATES DIFFERENCES IN PLASTICITY

It remains possible that genetic drift rather than adaptive divergence could cause the species‐specific differences in plasticity that we observed. However, given substantial divergence in leaf form and physiology under common garden conditions, the differences in plasticity of these same traits among species, and the higher fitness of each species at their native versus novel habitats, it seems much more likely that adaptive divergence between *S. aethnensis* and *S. chrysanthemifolius* is responsible for their distinct plastic responses (Taylor and Aarssen 1988; Emery et al. 1994; Donohue et al. 2001; Ho and Zhang 2018).

Different species can show differences in plasticity in laboratory or glasshouse experiments (Murren et al. 2014), but such differences are often difficult to connect to ecological divergence. For example, differences in plasticity between closely related species of *Phlox* could not be explained by ecology or relatedness (Schlichting and Levin 1984). However, by assaying plasticity for two closely related species using field transplant across the elevational gradients that include their natural habitats, we show that adaptive divergence, demonstrated by greater survival of the native species in their native habitats (**Fig**. **5**), is associated with the evolution of differences in plasticity. We also show that the magnitude and direction of plasticity in response to elevation depends on the trait being considered: the two species showed differences in the magnitude of plasticity across elevation in some traits (e.g., leaf complexity and SLA), but a contrasting pattern of plasticity in other traits (e.g., leaf indents and flavonol content). This suggests that adaptive divergence, even between closely related species, can change both the magnitude and direction of plasticity, which will depend on how selection varies in its effect on different traits (Via 1993).

Whether differences in plasticity promote adaptive divergence or arise during or after adaptive divergence remain a critical focus of research aiming to understand speciation, and also how populations will persist in novel environments (Hoffmann and Bridle 2021). If different populations of the common ancestor possessed differences in plasticity, then plasticity associated with local adaptation to the high elevation habitat could have created adaptive divergence, which then led to speciation (Reger et al. 2018). Alternatively, if the common ancestor possessed a large amount of genetic variation in plasticity, the high elevation habitat could have been colonised by genotypes with forms of plasticity better suited for survival there (the plasticity‐first hypothesis), with selection for them resulting in divergence in plasticity (West‐Eberhard 2005; Corl et al. 2018; Walter et al. 2020b).

Adapting to contrasting elevations is likely to maintain plasticity only in genes and traits important for coping with variation in the native environments of each species. Plasticity will therefore be reduced (or lost) for genes and traits that need to be continually expressed in their native habitat compared to their ancestral habitat. For example, *S. aethnensis* produces less complex (and glaucous) leaves that are, in contrast with *S. chrysanthemifolius*, effectively invariable across elevation (**Fig**. **6c,d**). However, adaptive divergence has likely favoured greater plasticity in traits critical for coping with the stressors experienced in each environment. For example, *S. chrysanthemifolius* has more complex leaves that show greater plasticity in leaf complexity across elevation (**Fig**. **6c,d**), probably to cope with extreme high temperatures at low elevation, as well as large daily and monthly fluctuations in temperature.

### ADAPTIVE AND NONADAPTIVE PLASTICITY IN NOVEL ENVIRONMENTS

When exposed to novel environments, each species is restricted to the plasticity that evolved in their native habitats, even if such plasticity is nonadaptive and reduces fitness. Our results suggest that although plasticity in *S. chrysanthemifolius* remains adaptive (at least to some extent) at novel high elevations, existing plasticity in *S. aethnensis* is nonadaptive at novel low elevations (**Fig**. **7**). This is consistent with the observation that *S. aethnensis* suffered greater mortality over summer immediately after the initial transplant, while *S. chrysanthemifolius* only suffered during winter and only at the highest elevation (**Fig**. **5**). Reduced survival at lower elevations in *S. aethnensis* was also associated with stronger reductions in leaf investment (**Fig**. **6g,h**) and lower photosynthetic activity (**Fig**. **6i**). Our results therefore suggest that *S. aethnensis* is specialised to the high‐elevation habitat and that rapid evolution will be necessary for this species to persist in novel environments created by climate change. These results are consistent with studies in other species that showed reduced plasticity in flowering time (Schmid et al. 2017) and morphology (Emery et al. 1994) in high‐elevation populations compared to low‐elevation populations. Such potential limits to adaptive plasticity could threaten the persistence of populations exposed to novel environments unless genotypic variation in plasticity (genotype × environment interactions) can allow rapid adaptation (Lande 2009; Chevin et al. 2010; Walter et al. 2020b). Further work needs to quantify genetic variation in plasticity, identify how selection (and changes in allele frequencies) affects the evolution of plasticity in different traits and environmental regimes (Schmitt et al. 1999; Pratt and Mooney 2013; McLean et al. 2014; Forsman 2015), and determine whether selection on plasticity could promote adaptation to novel environments (Lande 2009; Walter et al. 2020b).

### SPECIES DIFFERENCES IN GENE EXPRESSION WITHIN AND ACROSS ELEVATION

The native conditions experienced by any given species can determine how gene expression will change in response to environmental variation (Akman et al. 2016). We observed both differential expression between species as well as differences between species in gene expression changes along the elevational gradient. A large proportion of genes that differed in expression between the two species were differentially expressed across all elevations, suggesting inherent differences in gene expression that have evolved during speciation (**Fig**. **8a**). However, 7–23% (233–957 genes) of all genes that were differentially expressed between the two species were unique to any given elevation, which suggests that these two species show distinct patterns of gene expression even when exposed to the same environments.

Within a species, plasticity in gene expression in this experiment is represented by genes that are differentially expressed between their native elevation and other elevations. We found that about 50% of genes that were differentially expressed across elevational extremes showed strong changes in one species that were weaker or absent in the other species, again providing strong evidence that the two species have evolved substantial differences in plasticity (**Fig**. **9a**). However, only 121 genes (of about 4000) showed highly divergent patterns of gene expression between the species, characterized by strong overexpression in one species, but underexpression in the other species. This result suggests that species differences in expression profiles were dominated by differences in the magnitude of the response, rather than responses in opposite directions. Gene (co)expression networks were also broadly similar between the species (**Fig**. **9b**), although one network module was unique to *S. aethnensis* (**Fig**. **9c**) and was associated with responses to biotic stressors and changes in light conditions, which is consistent with evidence showing that changes in plasticity for specific gene networks can underlie adaptation (Kenkel and Matz 2016).

## AUTHOR CONTRIBUTIONS

JB, SH, GW, SCo, AC, and DF designed the study. GW, AC, DT, and SCa conducted the glasshouse and fieldwork. JC and BN extracted RNA and handled the transcriptome data. MP measured chlorophyll fluorescence, and VV grew plants and measured physiological differences between the two species. GW and JC analysed the data and wrote the manuscript with important contributions from all authors. All authors gave final approval for publication.

## CONFLICT OF INTEREST

The authors declare no conflict of interest.

### DATA ARCHIVING

Data from the common garden experiment and the 2017 and 2019 field transplants are found at https://doi.org/10.5061/dryad.9w0vt4bhb. Raw RNA reads from sequencing experiments have been uploaded to the Sequence Reads Archive (SRA) under the project number PRJNA603521.

Associate Editor: Dr. Matthew Walsh

Handling Editor: Dr. Andrew McAdam

## Supporting information

Supplementary material
